# Announcement

**Published:** 1896-07

**Authors:** 


### EDITORIAL.

The office of the Business Manager of THE JOURNAL is Nos. 311 and 312 Fitten Building.

The editors are not responsible for views expressed by contributors.

Articles for publication should reach this office not later than the 15th inst.

Address all communications, and make all remittances payable to THE ATLANTA MEDICAL AND SURGICAL JOURNAL, Atlanta, Ga.

Reprints of articles will be furnished, when desired, at a nominal cost. Requests for the same should always be made *on the manuscript.*

### ANNOUNCEMENT.

The managing editor of the JOURNAL is now absent in New York, engaged in post-graduate medical study. Until his return, and until proper notice appears in our columns, the editorial and literary department will be managed by Dr. A. W. Stirling, whom we take pleasure in commending to our readers as eminently qualified for the work which he has kindly agreed to assume in his absence. In Dr. Stirling's hands we know that the interests of this department of the JOURNAL will be conserved. L. B. G.

